# Involvement of Nucleotide-Binding Oligomerization Domain-Like Receptor Family Pyrin Domain Containing 3 Inflammasome in the Pathogenesis of Liver Diseases

**DOI:** 10.3389/fcell.2020.00139

**Published:** 2020-03-10

**Authors:** Congjian Shi, Hongqin Yang, Zhenghong Zhang

**Affiliations:** Provincial Key Laboratory for Developmental Biology and Neurosciences, Key Laboratory of Optoelectronic Science and Technology for Medicine of Ministry of Education, College of Life Sciences, Fujian Normal University, Fuzhou, China

**Keywords:** NLRP3 inflammasome, inflammation, non-alcoholic fatty liver disease, liver fibrosis, cirrhosis, hepatocellular carcinoma

## Abstract

The inflammasome is widely acknowledged for its crucial role in the pathogenesis of cancers and many neurodegenerative, metabolic, and auto-inflammatory diseases in recent years. Multiple types of inflammasomes exist. However, nucleotide-binding oligomerization domain-like receptor family pyrin domain containing 3 (NLRP3) inflammasome is the most often investigated inflammasome and has come to limelight in recent studies. NLRP3 inflammasome is a multi-protein complex. Its activation can cause the cleavage of inactive pro-caspase-1 into activated caspase-1, that ultimately promotes the transformation of pro-interleukin (IL)-1β and pro-IL-18 into biologically-active IL-1β and IL-18, respectively. These processes lead to the local inflammatory responses and induce pyroptosis, causing disparaging effects. Recently, numerous studies have shown that NLRP3 inflammasome plays an important role in the pathogenesis of liver diseases, including non-alcoholic fatty liver disease, liver fibrosis, cirrhosis, and hepatocellular carcinoma. Liver diseases have become a severe health burden worldwide, and there is adequate evidence indicating that the regulation of NLRP3 inflammasome acts as a guard against hazard to liver. In this review, we provide a straightforward overview of NLRP3 inflammasome as well as several frequent liver diseases. We then discuss the contribution and regulation of NLRP3 inflammasome during the pathogenesis of liver diseases, which may provide an important indication for the prevention and treatment of various liver diseases.

## Introduction

Currently, living conditions have greatly improved as compared to the earlier times. Humans consume considerably amount of nutrition, alcohol, and carbohydrate that upon combining with other endogenous and exogenous influential factors, aggravate liver burden ultimately leading to the lesions of liver. Liver plays an irreplaceable role in body metabolism, and liver diseases seriously affect normal human life. Common liver diseases including non-alcoholic fatty liver disease (NAFLD), liver fibrosis, cirrhosis, and hepatocellular carcinoma (HCC) are serious health threats to the people worldwide (Bouattour et al., 2018; Schuppan et al., 2018; Vonghia et al., 2019). Patients in the early stage of liver diseases usually have mild symptoms, which are often not felt by the patients. Thus, liver diseases are greatly concealed health dangers for humans worldwide. Paying attention to the pathogenesis of liver diseases and searching for treatments that are more efficient is thus, of utmost importance.

NLRP3 inflammasome has attracted considerable attention during recent years. It is basically a kind of multimeric protein complex which consists of NLRP3, apoptosis-associated speck-like protein containing a caspase activation and recruitment domain (ASC) and pro-caspase-1 (Chen et al., 2016; Wang et al., 2016, 2019). Latest studies indicate that the activity of NLRP3 inflammasome is closely related with diversified diseases, including rheumatoid arthritis (Wu et al., 2019), breast cancer (Ershaid et al., 2019), pleurisy (Yang S. et al., 2019), systemic lupus erythematosus (Tan et al., 2019), cardiovascular disease (Zhang Y. et al., 2019), and renal fibrosis (Guo et al., 2017). Till date, NLRP3 inflammasome is widely studied in liver diseases with its activity in liver diseases being comprehensively investigated, and numerous studies showing a key role of NLRP3 inflammasome in the pathogenesis of liver diseases, especially NAFLD, liver fibrosis, cirrhosis, and HCC (Wu et al., 2015; Yang et al., 2016; Wei et al., 2019). Consequently, there is a critical need to understand the effect of NLRP3 inflammasome in various kinds of liver diseases mentioned above that may be helpful in developing potential treatments to regain normal liver functions.

## NLRP3 Inflammasome

The inflammasome is one of the most important multimeric protein complexes that participate in immune system functions, and is widely known as an intracellular inflammatory machinery (Boini et al., 2014; Chen et al., 2015). It plays a pivotal role in recognizing infection, repairing damaged tissue and initiating the process of pathogen clearance (Strowig et al., 2012). The inflammasome is assembled by intra-cytoplasmic sensor protein called pattern recognition receptors (PRRs), adaptor protein ASC, and effector protein pro-caspase-1 (Fan et al., 2019). The types of PRRs present in it, which define the designation of inflammasome. The PRRs can perceive the pathogen-associated molecular patterns (PAMPs) and danger-associated molecular patterns (DAMPs), and then generate immune responses to eliminate infections and repair injured tissues (Bortolotti et al., 2018).

**FIGURE 1:**
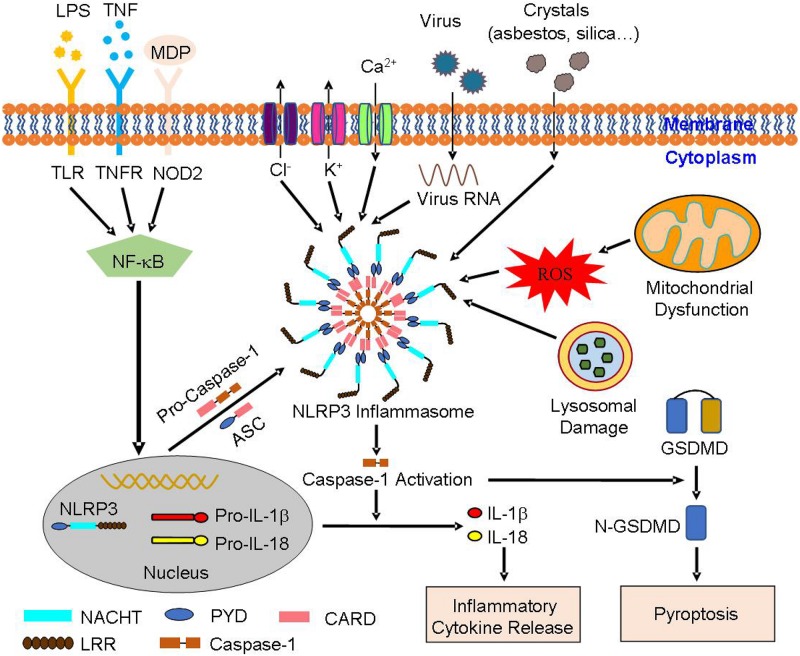
The structure and activation of NLRP3 inflammasome: NLRP3 inflammasome consists of NLRP3, ASC, and pro-caspase-1. A two-step mechanism leads to NLRP3 inflammasome activation, including the priming and the activation step. During the priming step, TLR, NOD2, or TNFR receives the stimulation induced by extracellular signaling molecules, leading to the activation of NF-κB. NF-κB then promotes the expression of NLRP3, pro-IL-1β, and pro-IL-18. During the activation step, the stimulation like K^+^ efflux, Ca^2+^ mobilization, Na^+^ influx, chloride efflux, ROS, mitochondrial dysfunction, and lysosomal damage leads to the assembly of NLRP3 inflammasome. In the process of NLRP3 inflammasome activation, activated caspase-1 transforms pro-IL-1β and pro-IL-18 into mature IL-1β and IL-18, resulting in the release of inflammatory cytokines. Further, activated caspase-1 dissociates GSDMD to release its N-terminus, form pores in the plasma membrane, and stimulate the occurrence of pyroptosis. ASC, apoptosis-associated speck-like protein containing a caspase activation and recruitment domain (CARD); GSDMD, gasdermin D; NF-κB, nuclear factor-κB; NLRP3, nucleotide-binding oligomerization domain (NOD)-like receptor family pyrin domain containing 3; pro-IL-1β, pro-interleukin-1β; pro-IL-18, pro-interleukin-18; ROS, reactive oxygen species; TLR, toll-like receptor; TNFR, tumor necrosis factor receptor.

Among multiple types of inflammasomes, NLRP3 inflammasome has received extra attention, with definitive studies on its function and structure. NLRP3 inflammasome is critical for immune defense, which can withstand bacterial, fungal, and viral infections (Man and Kanneganti, 2015). Cause the activation of NLRP3 inflammasome is an inflammatory process, it must be strictly regulated. In recent studies, a two-step mechanism has been proposed to explain the process (Swanson et al., 2019). The mechanism includes the priming step and the activation step (Figure 1). During the priming step, endogenous cytokines or microbial components in cell membrane stimulate toll-like receptor (TLR), nucleotide-binding oligomerization domain (NOD) 2 or tumor necrosis factor (TNF) receptor leading to the activation of nuclear factor-κB (NF-κB). NF-κB then promotes the synthesis of NLRP3 and the expression of pro-IL-1β and pro-IL-18 (Guo et al., 2016; Mathur et al., 2018). During the activation step, stimulation like K^+^ efflux, Ca^2+^ mobilization, Na^+^ influx, chloride efflux, reactive oxygen species (ROS), mitochondrial dysfunction, and lysosomal damage generate activation signals, leading to the assembly of NLRP3 inflammasome with caspase-1 mediating IL-1β and IL-18 excretion (Zhang et al., 2015; Kelley et al., 2019). IL-1β, primarily excreted by mononuclear macrophage cells, is an effective pro-inflammatory cytokine that recruits innate immune cells to the site of infection and regulates acquired immune cells. IL-18 stimulates the production of interferon-γ (IFN-γ) and enhances the ability of T cells and natural killer (NK) cells (van de Veerdonk et al., 2011). What’s more, capase-1 makes it possible for the non-conventional secretion of numerous cytosolic proteins (Mangan et al., 2018). In this process, activated caspase-1 also dissociates gasdermin D (GSDMD), and regulates the N-terminal domain of GSDMD by forming pores in the plasma membrane. It thus, stimulates an inflammatory form of cell death termed pyroptosis (An et al., 2019), which is persistently observed during microbial infections that combine the features of apoptosis and necrosis.

Accumulated evidences have already demonstrated that NLRP3 inflammasome participates in the pathogenesis of liver diseases like NAFLD, liver fibrosis, cirrhosis, and HCC. Plentiful existing literatures summarized the rough relationship between NLRP3 inflammasome and liver diseases, involved the changes of NLRP3 inflammasome components expression under different liver disease states, and knocked out the genes that play pivotal roles in the assembly process of NLRP3 inflammasome to observe the occurrence and development of liver diseases. However, a few literatures have pointed out the specific mechanism of NLRP3 inflammasome influencing the pathogenesis of liver diseases, more studies should be conducted to explore the definite underlying mechanism. And further studies focused on NLRP3 inflammasome activity may provide novel strategies for the treatments of these liver diseases.

## NLRP3 Inflammasome and NAFLD

### NAFLD Pathogenesis

NAFLD is one of the most common chronic liver diseases globally, which ranges from simple steatosis to steatohepatitis [non-alcoholic steatohepatitis (NASH)]. NAFLD is generally associated with insulin resistance, obesity, type 2 diabetes, and hyperlipidemia (Wan et al., 2016a). Approximately 20∼30% of people around the world are affected by NAFLD and its complications including a proportion of up to 75–100% obese people (Henao-Mejia et al., 2012). NAFLD is usually unnoticed by patients for its mild symptoms, therefore they are likely to neglect it (Shi et al., 2019). A “two-hit” hypothesis had been proposed to explain the pathogenesis of NAFLD. In the first hit, insulin resistance and lipid metabolism disorders lead to the excessive lipid deposition and increasing triglyceride (TG) in hepatocytes. This is followed by the second hit, which is a multifactorial process including mitochondrial dysfunction, lipid peroxidation, and excess production of ROS via oxidative stress (Zhu et al., 2018). These events thus, cause liver damage and without effective control measures, the damage may progress to grave diseases like liver fibrosis, cirrhosis, and even HCC. Despite extensive studies on NAFLD, there is still no approved pharmacological agent for treating it with people mainly adopting precautions such as weight-loss and healthy lifestyle to overcome the disease.

### NLRP3 Inflammasome in NAFLD

Recent studies have demonstrated that there is an intimate connection between NLRP3 inflammasome and NAFLD, with NAFLD mice showing an evident increased expression of NLRP3, ASC, and caspase-1 in comparison to the mice fed by normal diet (Lv et al., 2019), and a large proportion of mice NAFLD models are caused by high fat diets. An identical consequence was affirmed in HepG2 and L02 cells that showed that inhibition of NLRP3 activity decreases uric acid-induced lipid accumulation in both the cell lines (Wan et al., 2016a). Furthermore, the studies using NLRP3^–/–^, ASC^–/–^, and caspase-1^–/–^ mice suggest that the pathogenesis of NAFLD is associated with the activation of NLRP3 inflammasome (Henao-Mejia et al., 2012). Moreover, TNF-α level are evidently higher in NAFLD mice than that in normal mice. Mitsuyoshi et al. (2017) have reported that liver NLRP3, IL-1β, IL-18, and pro-caspase-1 mRNA levels are significantly increased in NAFLD patients than those in healthy people, suggesting that NLRP3 inflammasome activation may promote the pathological progression of NAFLD. Chitturi and Farrell have reported that insulin resistance is a pivotal pathogenic factor associated with NAFLD, while inflammatory factors including IL-1β, IL-18, and NF-κB have a close connection with insulin resistance. Moreover, the activation of these inflammatory factors is regulated by the assembly of NLRP3 inflammasome (Chitturi and Farrell, 2001). Under NAFLD state, hepatocytes are subjected to excessive oxidative stress; hence, the generation of excess ROS is inevitable. ROS as an intracellular danger signal makes thioredoxin interacting protein (TXNIP) to dissociate from thioredoxin (TRX), thus triggering the activation of NLRP3 inflammasome. Studies also indicate that TXNIP upregulation leads to the secretion of IL-1β and IL-18 (Zheng et al., 2018). In a NASH model, mice with IL-1α and IL-1β gene knockout noticeably prevented the pathological process from steatosis to steatohepatitis, while IL-1β increases TG level in hepatocytes and induces hepatocytes death by collaborating with TNF-α (Wan et al., 2016b). All these evidences imply that NLRP3 inflammasome plays an important role in NAFLD, but the definite underlying mechanism remain elusive. Our team has a decent foundation for investigating NLRP3 inflammasome (Zhang Z. et al., 2019), and the steatosis models of mice livers and L02 cells were successfully established in our previous study (Shi et al., 2019), we intend to further explore the important role of NLRP3 inflammasome in the pathological process of NAFLD and detect the potential mechanism.

**FIGURE 2:**
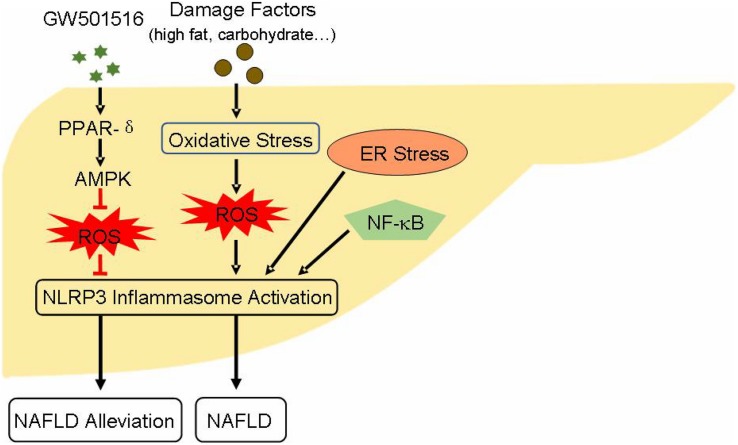
Effect and activity regulation of NLRP3 inflammasome during the pathogenesis of NAFLD: PPAR-δ agonist GW501516 activates PPAR-δ through AMPK/ROS pathway, suppresses NLRP3 inflammasome activation, which ultimately alleviates NAFLD status. Danger factors like high fat and carbohydrate give rise to hepatocyte oxidative stress. This process generates excess ROS leading to the activation of NLRP3 inflammasome. Moreover, NF-κB signaling and ER stress also induce NLRP3 inflammasome activation. These influential factors ultimately aid the progression and pathogenesis of NAFLD. AMPK, AMP-activated protein kinase; ER, endoplasmic reticulum; NAFLD, non-alcoholic fatty liver disease; PPAR-δ, peroxisome proliferator-activated receptors-δ.

AMP-activated protein kinase (AMPK) activation has been proved to alleviate NAFLD state. It has demonstrated that the activation of peroxisome proliferator-activated receptors-δ (PPAR-δ), a kind of nuclear receptor protein, restrains the decline of AMPK expression and increases the phosphorylation of AMPK-α (Lee H.J. et al., 2015). Moreover, AMPK activation inhibits the expression of IL-1β and caspase-1, and suppresses ROS generation (Lee H.J. et al., 2015), and AMPK/ROS pathway is closely related with the activation of NLRP3 inflammasome. The activation of NF-κB-related signaling pathway also has an accelerating effect in the pathogenesis of NAFLD (Xiao et al., 2018), while NF-κB engages in the priming step of NLRP3 inflammasome activation. Other studies have shown that endoplasmic reticulum (ER) stress can induce the activation of NLRP3 inflammasome (Han et al., 2018), ultimately leading to hepatocyte death and NAFLD (Figure 2). Studies that aim at understanding the regulation of NLRP3 inflammasome may provide new potential strategy for the treatment of NAFLD. Resveratrol, a polyphenol widely present in grapes, pomegranates, blueberries, and mulberries, is reported to ameliorate NAFLD by suppressing the activation of NLRP3 inflammasome (Yang and Lim, 2014). A natural product such as honey, which made by bees, can protect liver during the progression of NAFLD through restraining NLRP3 inflammasome activity (Xiao et al., 2016). There are many similar substances, such as sulforaphane, salvianolic acid A, MCC950, andrographolide, silybin, salidroside, etc. That can be assessed for their effects on NLRP3 inflammasome. Since NLRP3 inflammasome is crucial for the pathogenesis of NAFLD and the initiation of liver inflammatory responses, suppressing its endogenous activation may have a favorable effect in improving NAFLD.

## NLRP3 Inflammasome and Liver Fibrosis/Cirrhosis

### Liver Fibrosis/Cirrhosis

Liver fibrosis is a wound healing process that occurs in the liver. It usually results from sustained, chronic liver injury, including viral hepatitis, long-term drinking, autoimmune diseases, and NAFLD (Seki and Brenner, 2015). In absence of intervention, liver fibrosis ultimately develops into cirrhosis with time and is characterized by extracellular matrix (ECM) substituting the bulk of liver parenchyma. The ECM can increase up to 10-fold in cirrhosis compared with liver fibrosis (Schuppan et al., 2018), and cirrhosis is the final manifestation stage of liver fibrosis. For patients with advanced cirrhosis, liver transplantation is the only effective treatment. It is now widely accepted that the occurrence and development of liver fibrosis is tightly related with the activation of hepatic stellate cells (HSCs). HSCs in healthy liver can be detected between hepatocytes and sinusoidal endothelium. They are quiescent and function to store retinoids and generate glial fibrillary acidic protein (GFAP). However, sustained chronic liver injury causes the activation of HSCs and transforms them into myofibroblasts, while Kupffer cells are also activated in this process (Wang et al., 2018). Except HSCs, others cells with the capability of developing into myofibroblasts include endothelial cells, epithelial cells, biliary duct fibroblasts, mesenchymal cells, and mesangial cells (Artlett and Thacker, 2015). Myofibroblasts accumulate in fibrotic liver while they are rarely detected in healthy liver. Hence, they are considered as the principle effector cells of liver fibrosis. Moreover, myofibroblasts synthesize interstitial or fibrillar ECM, also referred to as scar tissue in liver parenchyma. The deposition of ECM is the morphological characteristic of liver fibrosis (Koyama et al., 2016). Chemokines, cytokines, growth factors, oxidative stress, and dominant ECM (mainly types I and III collagens) contribute in the pathogenesis of liver fibrosis (Sanchez-Valle et al., 2012). At present, the studies on the potential treatments of liver fibrosis primarily aim at preventing or reducing liver injury, transferring bone-marrow derived restorative macrophages or inhibiting inflammatory monocyte infiltration, promoting myofibroblasts apoptosis, and accelerating ECM degradation (Schuppan and Kim, 2013; Lee Y.A. et al., 2015).

### NLRP3 Inflammasome in Liver Fibrosis/Cirrhosis

Accumulated evidences indicate that NLRP3 inflammasome plays an important role in the pathogenesis of liver fibrosis/cirrhosis. In the pathological process of liver fibrosis, the most discussed phenomenon is the activation of HSCs. The activation process is accompanied by increased levels of ECM, α-smooth muscle actin (α-SMA), transforming growth factor-β (TGF-β), platelet-derived growth factor (PDGF), and tissue inhibitor of metalloproteinase 1 (TIMP1) (Koyama et al., 2016). Studies have shown that wild type mice develop more severe liver fibrosis induced by carbon tetrachloride (CCL_4_) as compared to the NLRP3^–/–^ and ASC^–/–^ mice, as evidenced by higher α-SMA expression and more Sirius Red staining area in the wild-type than in the knockout mice (Watanabe et al., 2009). Conversely, the activation of NLRP3 inflammasome in mice facilitates the pathological process of liver inflammation and fibrosis (Inzaugarat et al., 2019). Activated caspase-1, IL-18, and IL-1β signaling are also pivotal to the development of liver fibrosis resulting from the activation of NLRP3 inflammasome. Both IL-1β and IL-18 can induce the deposition of collagen (Artlett, 2012). MCC950, an agent that can block NLRP3 inflammasome, decreases the expression of liver caspase-1 and the numbers of macrophages and neutrophils, ultimately alleviating liver fibrosis (Mridha et al., 2017). These studies provide overwhelming evidences about the association of NLRP3 inflammasome activation with accelerated liver fibrotic pathological process.

The renin–angiotensin system (RAS) possesses regulatory effects on liver fibrosis. Angiotensin II (Ang II), a key player in RAS, induces the generation of NADPH oxidase 4 (NOX4)/mitochondria-derived ROS and aggravates liver fibrosis, while NLRP3 inflammasome activation is tightly connect with NOX4/mitochondria-derived ROS (Cai et al., 2016). The inhibition of Ang II alleviates liver fibrosis by reducing the generation of Ang II-induced ROS and influencing NLRP3 inflammasome activation. In addition, Ang II stimulation induces ECM deposition and the proliferation of HSCs (Ning et al., 2017; You et al., 2019). Furthermore, crystalline monosodium urate is known to be an activator of NLRP3 inflammasome *in vitro*, that increases TGF-β and collagen I expression in LX-2 cells (immortalized human HSCs) and primary mouse HSCs (Watanabe et al., 2009). NF-κB signaling is also important for the pathological process of liver fibrosis, while NLRP3 inflammasome activation is accompanied by the upregulation of NF-κB signaling. Additionally, suppressing NF-κB signaling along with the promotion of HSCs apoptosis improves liver fibrosis (Zheng et al., 2019). These factors are intimately related to the activation of NLRP3 inflammasome, prospectively, it will be a splendiferous choice to prevent and treat liver fibrosis by probing the inhibition of NLRP3 inflammasome activity.

## NLRP3 Inflammasome and HCC

### Hepatocellular Carcinoma

Hepatocellular carcinoma is the most common liver malignancy, accounting for more than 90% of liver cancers, and a major cause of cancer-related mortality (Zhu et al., 2019). The situation of HCC is getting austere with its morbidity and mortality increasing year by year. Men are confronted with approximately threefold higher risk than women, on account of higher likelihood to smoking and drinking (El-Serag and Kanwal, 2014). Main risk factors of HCC are closely related to hepatitis B virus (HBV), hepatitis C virus (HCV), alcoholic liver disease (ALD), NAFLD, liver fibrosis, obesity, diabetes, and smoking (Inchingolo et al., 2019). Most cases of HCC are detected at advanced stages and less than 40% of HCC patients have the chance to be treated surgically (Forner et al., 2012). Therefore, the diagnosis of HCC becomes particularly important. Current methods for evaluating HCC include histological observation, serum alpha-fetoprotein (AFP) level detection, computed tomography (CT), and magnetic resonance imaging (MRI) (Ayoub et al., 2019). The major clinical methods used in the treatment of HCC include surgical resection, liver transplantation, ablative therapies, chemoembolization, and systemic therapy (Akateh et al., 2019). Although many new therapeutic schedules are applied in clinical settings, the 5-year survival rate is typically unfavorable in addition to adverse reactions and drug resistance associated with the use of anticancer drugs (Yang H. et al., 2019). Consequently, it is extremely necessary to find new targets for the treatment of HCC.

### NLRP3 Inflammasome in HCC

As HCC is one of the foremost health hazards, increasing number of studies have been devoted for it. NLRP3 inflammasome has been demonstrated to play a significant role in NAFLD and liver fibrosis/cirrhosis. Hence, increasing attention has been endowed on understanding the regulation of NLRP3 inflammasome activity in the pathogenesis of HCC. However, the underlying mechanisms remain greatly controversial. Expression of NLRP3, ASC, caspase-1, and IL-1β are significantly decreased in HCC tissues as compared to the corresponding non-cancerous liver tissues. Moreover, the expression of these genes also decreases progressively with the development of pathological and clinical stage of HCC (Wei et al., 2014). These results imply that the activation of NLRP3 inflammasome has protective effects in the pathogenesis of HCC. Taking into account the gender difference in the incidence of HCC, 17β-estradiol (E_2_), and estrogen receptor (ER) β have received extra consideration. Studies have shown that the expression of ERβ also decreases in the HCC tissues. Wei et al. (2015) have reported that HCC cell lines (SMMC7721, BEL7402, and HepG2 cells) treated with E_2_ -activated NLRP3 inflammasome through E_2_/ERβ/MAPK signaling, eventually ameliorated HCC progression. Furthermore, the NLRP3 inflammasome activated by E_2_ treatment -induced caspase-1-dependent pyroptosis in HepG2 cells, led to the suppression of HCC progression (Wei et al., 2019).

However, there are contrary opinions about the influence of NLRP3 inflammasome activation in the pathogenesis of HCC. Luteoloside, a natural flavonoid with many pharmacological activities, suppresses the NLRP3 inflammasome activation and induces the inhibition of proliferation, invasion, and metastasis in HCC cells (Huh7 and SMMC7721) (Fan et al., 2014). Wan et al. (2018) have reported that microRNA-223-3p promotes apoptosis and inhibits the proliferation in Hep3B cells by suppressing the activation of NLRP3 inflammasome. These results suggest that NLRP3 inflammasome activation accelerates the pathological process of HCC. Thus, inhibiting the activity of NLRP3 inflammasome may become a novel potential target for treating HCC. The relationship between NLRP3 inflammasome and HCC has not been thoroughly studied yet, we need to make clear the definite molecular mechanism by which NLRP3 inflammasome affects the pathogenesis of HCC. Additional studies are essential to understand the relationship between NLRP3 inflammasome and HCC progression in future.

## Summary

**FIGURE 3:**
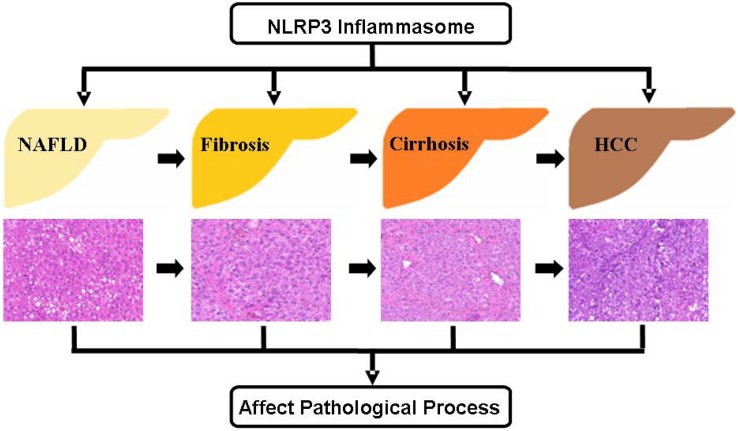
Role of NLRP3 inflammasome during the different stages of liver diseases NAFLD is the most common form of chronic liver diseases, which can progress to more serious diseases, including liver fibrosis, cirrhosis, and HCC. The regulation of NLRP3 inflammasome activity has significant effect on the pathogenesis of these liver diseases. HCC, hepatocellular carcinoma.

Liver diseases have been considered as grave health hazards globally. Current studies have clearly shown that NAFLD has a high incidence in the population, and without effective interventions, it can progress toward liver fibrosis, cirrhosis, and eventually HCC (Figure 3). During the pathogenesis of NAFLD, liver fibrosis, and cirrhosis, the activation of NLRP3 inflammasome accelerates the pathological progression aggravating the symptoms of liver diseases on human body. In contrast, the impact seems to be reverse in HCC. Recent studies have shown that NLRP3 inflammasome activation acts as a defender in the pathogenesis of HCC. On the other hand, some other studies have indicated the reverse in case of HCC. Thus, it is worthwhile to explore more precisely the role of NLRP3 inflammasome in HCC as this area still has many controversies. Undoubtedly, there is no denying that NLRP3 inflammasome plays a critically function in the pathogenesis of liver diseases and further investigations aimed to delineate the effects of NLRP3 inflammasome in liver diseases are imperative. Fortunately, the discovery and exploration of NLRP3 inflammasome has provided a novel insight into the liver diseases, which may be further exploited as an important target for the prevention and treatment of liver diseases.

## Author Contributions

CS wrote the manuscript draft. CS, HY, and ZZ revised it. All authors read and approved the final version of the manuscript for publication.

## Conflict of Interest

The authors declare that the research was conducted in the absence of any commercial or financial relationships that could be construed as a potential conflict of interest.
